# Extracts of Adipose Derived Stem Cells Slows Progression in the R6/2 Model of Huntington's Disease

**DOI:** 10.1371/journal.pone.0059438

**Published:** 2013-04-02

**Authors:** Wooseok Im, Jaejun Ban, Jiyeon Lim, Mijung Lee, Soon-Tae Lee, Kon Chu, Manho Kim

**Affiliations:** Department of Neurology, Biomedical Research Institute, Seoul National University Hospital, Seoul, Korea; Oregon Health & Science University, United States of America

## Abstract

Stem cell therapy is a promising treatment for incurable disorders including Huntington's disease (HD). Adipose-derived stem cell (ASC) is an easily available source of stem cells. Since ASCs can be differentiated into nervous stem cells, it has clinically feasible potential for neurodegenerative disease. In addition, ASCs secrete various anti-apoptotic growth factors, which improve the symptoms of disease from transplanted ASCs. Thus, cell-free extracts of ASCs (ASCs-E) could be a potential candidate for treatment of HD. Here, we investigated effects of ASCs-E on R6/2 HD mouse model and neuronal cells. In R6/2 HD model, injection of ASCs-E improved the performance in Rotarod test. ASCs-E also ameliorated striatal atrophy and mutant huntingtin aggregation in the striatum. In Western blot increased expressions of p-Akt, p-CREB and PGC1α were noted by injection of ASCs-E, when comparing to the R6/2 HD model. Neuro2A neuroblastoma cells treated with ASCs-E showed increased expression of p-CREB and PGC1α. In conclusion, ASCs-E delayed disease progression in animal model of HD by restoring of CREB-PGC1α pathway and could be a potential resource for treatment of HD.

## Introduction

Huntington's disease (HD) is an autosomal dominant neurodegenerative disorder characterized by loss of striatal projection neurons. HD patients undergo involuntary motor movement, psychiatric manifestation and cognitive decline [1]–[4]. The huntingtin gene encodes a protein called huntingtin and HD patient has an expanded CAG repeats in exon 1 of the huntingtin gene. These CAG repeats express polyglutamine and this protein repeat is related with the aggregation of mutant huntingtin in neuron, which causes the cell death of striatum in HD [1], [2], [4], [5]. There is ongoing research for a cure for HD, however only a few medications can relieve symptoms in HD [3], [6].

Autologous transplantation of stem cells has been suggested as an ideal source for cell therapy, since problem of immune rejection is less likely. Among the tissue derived stem cells, adipose stem cell (ASC) is a feasible source for stem cell-based therapy, considering their abundance, multipotency, and ethical consideration [7]–[10]. ASCs transplantation in vivo models have been reported to be beneficial. However, whether transplanted ASCs replaced damaged cells is unclear [11], [12]. Stem cells secrete various factors which can modulate a hostile microenvironment of diseases [13]–[16]. ASCs also express multiple growth factors and could be used to treat diseases via secretion factors, called paracrine mechanism [10], [17]–[19].

One of the pathomechanisms of HD progression is transcriptional and mitochondrial dysregulation induced by mutant huntingtin (mHtt) [4], [20]. In HD, peroxisome proliferator-activated receptor γ coactivator-1α (PGC-1α), which affects mitochondrial energy metabolism, is known to be imbalanced by mHtt and, thus accelerating cell death [21]–[23]. Expression of PGC-1α is known to be controlled by cAMP response element binding protein (CREB) [24] which is also altered in HD [22], [25].

In this study, we attempted to test whether or not the extracts of ASCs (ASCs-E) can be an alternative to direct ASCs transplantation. To investigate effects of ASCs-E in HD transgenic mice, ASCs-E was intraperitoneally injected and changes of disease progression were monitored. We also examined whether ASCs-E could modulate Akt, CREB and PGC-1α expression in brain, which is imbalanced in HD. To confirm the effects of cell-free ASCs-E in vitro, neuro2A (N2A), mouse neuroblast cell, was treated with ASCs-E and examined p-Akt, p-CREB and PGC-1α expression.

## Materials and Methods

### Ethics Statement

Subcutaneous adipose samples were obtained from a normal human who provides the written informed consent to participate in this study and Institutional Review Board in Seoul National University Hospital approved this study. All animal studies were carried out with the approval of the Institutional Animal Care and Use Committee (IACUC) of Seoul National University Hospital, which was accredited by the Association for the Assessment and Accreditation of Laboratory Animal Care International.

### Preparation of human ASCs-E

The adipose tissues were minced and digested in 0.075% collagenase type I solution (Invitrogen, USA) in phosphate-buffered saline (PBS) with gentle agitation for 1 hour at 37°C. Digested tissues were centrifuged at 200×g for 10 minutes and upper mature adipocyte fractions were removed from stromal fractions. The remaining stromal fractions (pellet) were treated with red blood cell lysis buffer (Sigma, USA) for 10 minutes at room temperature, filtered through a 100–μm nylon mesh, and centrifuged at 200×g for 10 minutes. The combined stromal fractions of the samples were re-suspended and cultured in endothelial growth medium –2 MV (EGM–2 MV; Lonza, USA). For the preparation of human ASCs – E, the cultured ASCs were harvested in cold PBS and centrifuged at 200×g for 5 minutes. The ASCs were suspended with an extraction buffer (1 mM DTT, 1 mM EDTA, protease inhibitor cocktail P8340, 0.1% DEPC in PBS), and lysed by several mild mechanical air pressure. The lysate was centrifuged at 14,000×g for 15 minutes, and the supernatant was passed through a syringe filter unit (0.45 μm). Dulbecco's modified Eagle's medium (DMEM) (Welgene, Korea) was used as extraction buffer for in vitro treatment.

### Neuro2A culture and ASCs-E treatment

N2A cells were seeded onto 6-well culture plates (SPL, Korea) with DMEM containing 10% fetal bovine serum. Two days later, N2A cells were exposed to 10 μg/ml of ASCs-E for 1day. Following incubation with ASCs-E, cells were harvested and lysed in Radio Immuno Precipitation Assay (RIPA) buffer (Thermo, USA) with freshly added protease inhibitor and phosphatase inhibitor (Roche, USA). After cellular debris was cleared by centrifugation, protein concentration was determined by the BCA protein assay (Pierce, USA) according to the manufacturer's instructions.

### Huntington's disease animal model and ASCs-E injection schedule

We used transgenic HD mice of the R6/2 line (B6CBA-Tg(HDexon1)62Gpb/3J, 111 CAGs) and their WT littermates (Jackson Laboratories, USA). The R6/2 transgenic mouse model expresses exon 1 of a human mHtt and is the most widely used animal model for studying HD [5]. These mice were obtained by crossing ovarian transplant hemizygote females with B6CBAF1/J males. The mice were housed in groups with ad libitum access to food and water and a 12 hours light/12 hours dark cycle. The genotype was assessed using a PCR assay.

In R6/2 mice, disease phenotype appears at 8 weeks of age. Considering protective effect of ASCs-E, injection was started at 6 weeks old. ASCs-E was injected intraperitoneally two times a week (40 mg/kg) and control mice were injected with vehicle (equal volume of extraction buffer). The ASCs-E was freshly prepared just before administration and the injection dosage was selected according to our previous published reports [26], [27].

### Rotarod performance and weight measurement

**Figure 1:**
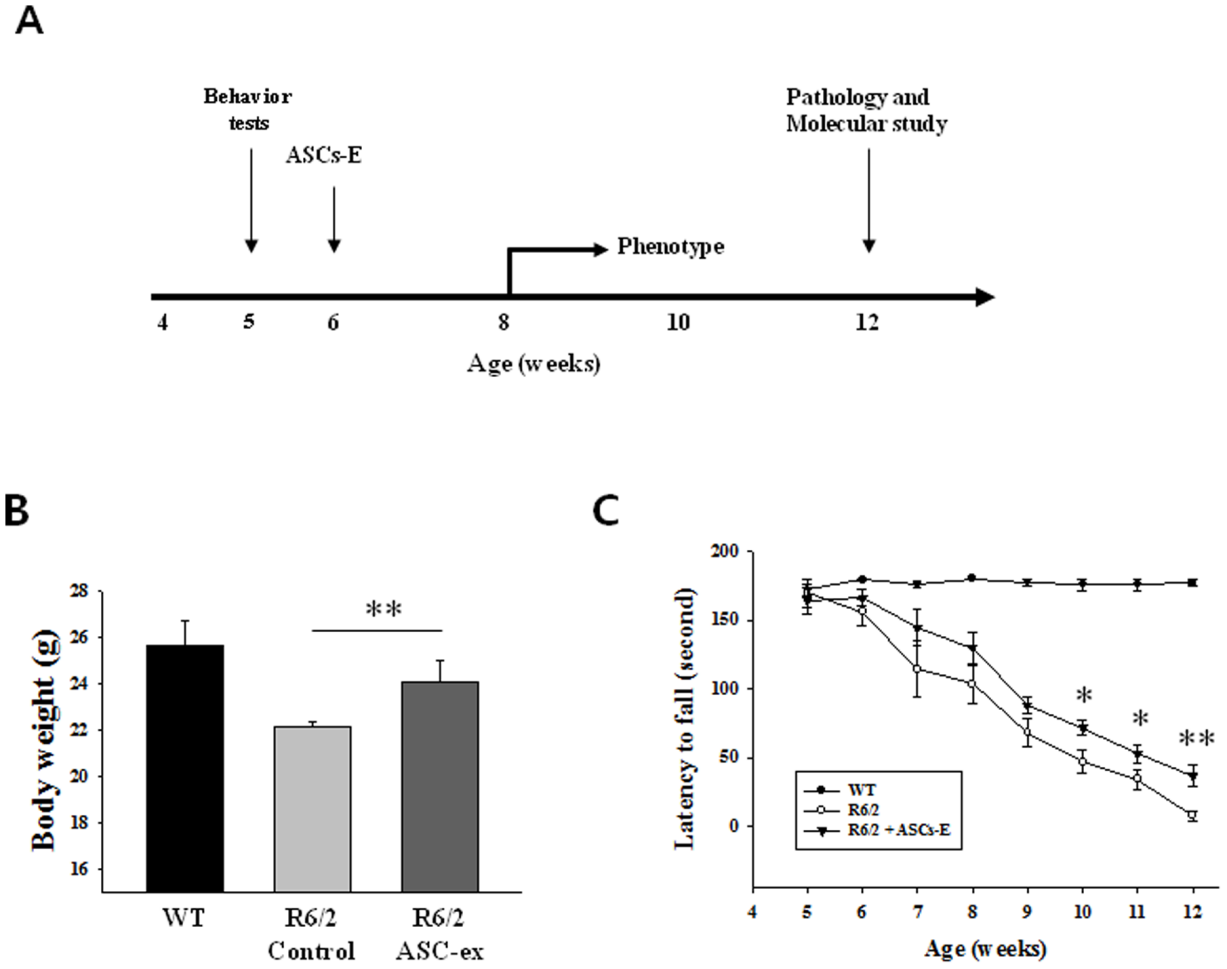
Experimental schedule and disease phenotypes of R6/2 mice. (A) Schedule for ASCs-E injection, behavior test, weight measure and brain sampling. (B) ASCs-E injection mitigated weight loss in R6/2 mouse at 12 weeks age. (C) Rotarod test showed better motor performance at 10, 11 and 12 weeks of age in ASCs-E treated R6/2 compared with control. * *p*<0.05, ** *p*<0.01.

The Rotarod test was evaluated using a rotarod apparatus (Jungdo Instruments, Korea). Mice were placed on the rod with an accelerating rotating speed from 4 to 40 rpm over a period of 3 minutes with a 15 minutes rest between trials. Mice were trained on three consecutive days for three trials per day at 4 weeks of age. Three trials were performed and the mean latencies to fall were used to analyze data. Rotarod evaluation and weight measurement were performed every week from 5 to 12 weeks (Figure 1A).

### Immunohistochemistry and striatal volume analysis

For immunohistochemistry, mice were anesthetized and perfused through the heart with 10 ml of cold saline and 4% paraformaldehyde in 0.1 M PBS at 12 weeks of age. Brains were removed from the skull, cryoprotected in 30% sucrose solution at 4°C, and sectioned 20 μm. Free-floating sections were washed and blocked with normal goat serum, then stained with the EM48 mHtt antibody (1∶300; Millipore). On the following day, the sections were washed in PBS with three times and incubated with FITC conjugated anti-rabbit IgG (1∶50; Jackson ImmunoResearch Laboratories) for 2 hours.

To examine the volume of striatum in R6/2 mice, serial-cut 200-μm coronal tissue sections from the rostral segment of the neostriatum to the level of the anterior commissure (bregma 1.54 to 0.10 mm) were used for Nissl staining. The areas of the striatum and the peristriatum were defined from each serial section, and gross volumes were measured with integrating each sectional area using Image-Pro Plus (Media Cybernetics, USA).

### Protein extracts and western blot analysis

Brains of R6/2 mice were isolated, immediately frozen on liquid nitrogen, and stored at −70°C until protein extraction. Cultured N2A cells were washed and harvested in PBS using a cell scraper. Protein extracts were prepared using RIPA buffer (Thermo, USA) with freshly added protease inhibitor and phosphatase inhibitor (Roche, USA). 30 μg protein samples were separated on 10% SDS-PAGE and transferred onto PVDF membrane. The blots were probed with primary antibodies: PGC-1α (1∶1000; Santa Cruz, CA), p-Akt (1∶1000; Cell Signaling), CREB (1∶1000; Cell signaling), EM48 (1∶200; Millipore), followed by horseradish peroxidase conjugated secondary anti-mouse or rabbit antibody (1∶5000; GE Healthcare), and blots were developed using ECL. Western blots were scanned and intensity values were obtained using ImageJ software.

### Statistical analysis

All values shown in the figures are presented as mean ± standard error. Rotarod performance and weights were analyzed by ANOVA with Fisher's post hoc test at each week of age. Western blot and histological results were analyzed by Student's t-test. A 2-tailed probability value below 0.05 was considered statistically significant. Data were analyzed using SPSS version 17.0 (SPSS Inc., USA).

## Results

### ASCs-E slowed behavioral phenotypes of R6/2 mice model

To investigate effects of ASCs-E on behavioral deficits of R6/2 mice, ASCs-E was injected from 6 weeks old and phenotypes were examined. Mice showed gradual weight loss from 10 to 12 weeks of age. In contrast with vehicle treated R6/2 mice, ASCs-E injected R6/2 showed delayed progression of weight loss at 12 weeks old (22.1±0.2 vs. 24±0.9, p<0.01) (Figure 1B). In rotarod test, ASCs-E treated group showed lowered latency to fall compared with vehicle treated group 10, 11 (p<0.05) and 12 weeks (p<0.01) of age (Figure 1C).

### ASCs-E reduced striatal atrophy and mutant Htt aggregation of R6/2 mice

**Figure 2:**
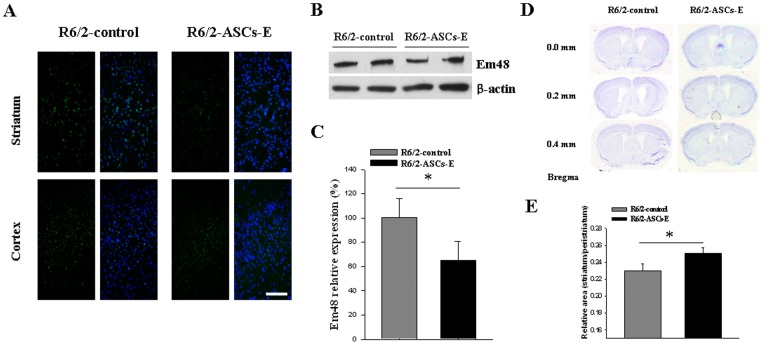
Striatal atrophy and mHtt aggregation. (A) Striatal mHtt aggregation was mitigated in ASCs-E treated group. (B) mHtt aggregation was visualized by western blot. (C) mHtt aggregation level was higher in ASCs-E treated R6/2 mouse brain compared with R6/2 control. (D) Striatum was sectioned and stained with Nissl in R6/2 mouse treated with ASCs-E or vehicle. (E) Striatum/peristriatum ratio is higher in ASCs-E injected group compared with vehicle treated group. Bar  = 100 μm, * *p*<0.01.

R6/2 mouse shows striatal atrophy and mHtt aggregation in striatum and cortex during disease progression [28]–[30]. To examine histological changes of the brain, volume of striatum and mHtt aggregation were evaluated at 12 weeks of age. To evaluate effect on mHtt aggregation, brains were sectioned and sliced tissues were stained with an EM48 antibody which detects aggregation of mutant huntingtin. As shown in figure 2A, injection of ASCs-E reduced mHtt aggregation in the striatum but not in the cortex. We extracted proteins from R6/2 mouse brain and mHtt aggregation was measured by western blot. ASCs-E treated group showed decreased mHtt aggregation in the brain compared with vehicle treated group (Figure 2B, C). Volumes of striatum and peristriatum were measured respectively and the ratio of striatum to peristriatum was calculated. We found that ASCs-E injected R6/2 brain showed an increased ratio of striatum to peristriatum compared with vehicle injected R6/2 brain (0.22±0.01 vs. 0.25±0.01, p<0.01, n = 7) (Figure 2D, E).

### ASCs-E activates CREB and PGC-1α in R6/2 mice and neuronal cells

**Figure 3:**
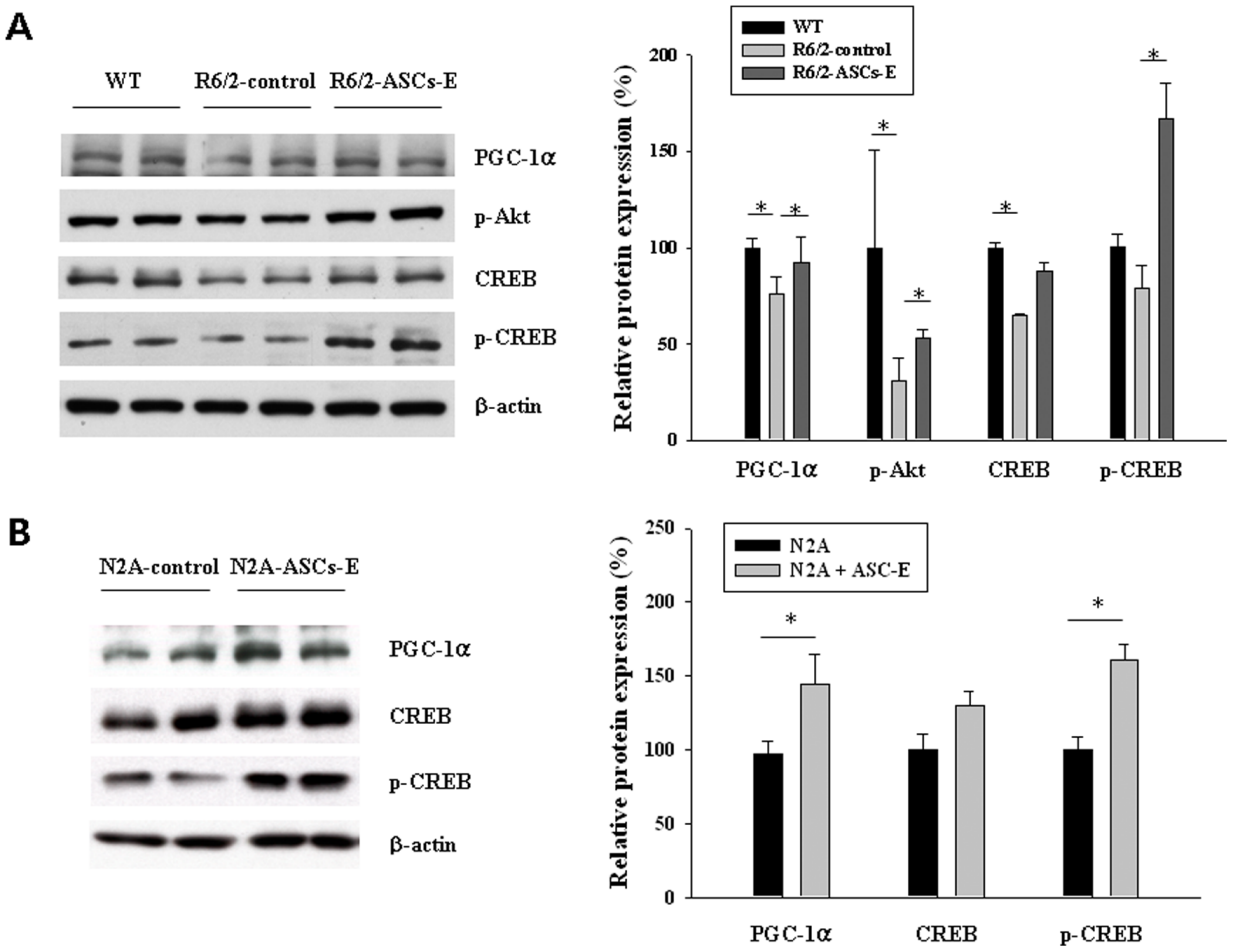
Restoration of p-Akt, CREB and PGC-1α by ASCs-E. (A) Western blot analysis confirmed that upregulation of PGC-1α, p-CREB and p-Akt in ASCs-E treated R6/2 mice brain and (B) PGC-1α and p-CREB in ASCs-E treated neuro2A cells. Bar graphs show the relative levels of protein expressions normalized to β-actin. * *p*<0.05.

Dysfunction of CREB-PGC-1α pathway has been regarded as the key molecules for HD progression [22], [25]. To examine effects of ASCs-E injection on this pathway, 12 weeks old R6/2 mice treated with control vehicle or ASCs-E for 6 weeks were sacrificed and western blot analysis was performed. Transplantation of ASCs-E promoted expression of p-Akt, p-CREB and PGC-1α (p<0.05 vs. R6/2 control), whereas they were decreased in R6/2 control mice (Figure 3A). To examine in vitro effects of ASCs-E, neuro2A cells were cultured and incubated with ASCs-E for 1 day. Then cells were harvested and levels of p-Akt, p-CREB and PGC1α were measured by western blot. In ASCs-E treated cells, levels of p-CREB and PGC1α were significantly increased (Figure 3B). Overall, neuroprotective p-CREB-PGC1α pathway was activated by treatment of ASCs-E.

## Discussion

In this study, we investigated whether treatment of ASCs-E affects expression of HD related molecules in neuronal cell line and disease progression in R6/2 transgenic mouse model. In neuronal cells, we observed that ASCs-E upregulates expression of p-CREB and PGC-1α, which could modify HD progression. We found that ASCs-E reduced mHtt aggregate and striatal atrophy in the brain of R6/2 mice, and slowed progression of HD phenotype, such as weight loss or decline of rotarod performance. We observed that lowered activation of Akt-CREB-PGC-1α pathway was recovered by injection of ASCs-E. Our study suggests that systemic injection of ASCs-E could slow ongoing HD progression.

Recent studies reported that mitochondrial dysfunction caused by imbalance of CREB-PGC-1α pathway is critically involved in HD progression. Inhibition of CREB activity causes PGC-1α impairment, inducing cell death by glutamate toxicity in striatum in HD [21]–[23], [25]. Considering that activation of PGC-1α protects against mitochondrial dysfunction and cell toxicity induced by mHtt, activation of CREB-PGC-1α pathway can have a key role to inhibit the progression of HD. P-Akt is believed to implicate CREB-PGC-1α expression and HD progression [31], [32]. In this study, we found ASCs-E activates Akt, CREB and PGC-1α, indicating that therapeutic effects of ASCs-E may be from upregulation of neuroprotective CREB-PGC1α pathway.

There are concerns about adverse effects in cell transplantation such as a risk of microvascular embolism or teratoma formation [33]. However, stem cell can have paracrine effects on a disease related hostile microenvironment. For example, stem cells could alleviate various diseases by modulating host environment such as immune regulation and neuroprotection via paracrine mechanism [10], [13]–[19]. Growth factors secreted by ASCs include EGF, FGF, VEGF, HGF and IGF-1, implying that these cells are sufficient for paracrine effect [10], [17]–[19]. These growth factors are believed to exist in the brain and exert important functions in neuronal cell development, survival and maintenance [34]. Injection of ASCs improved symptoms of R6/2 mice, suggesting the paracrine effects of ASCs as one of the underlying mechanisms in cell transplantation [18]. In this study, ASCs-E was beneficial on HD, similarly to ASCs injection, confirming the paracrine effects of ASCs.

To use ASCs-E clinically, however, further researches are required to elucidate some issues. For instance, we do not know what molecule of ASCs-E exert therapeutic effects. Previous reports showed that therapeutic effect of ASCs-E disappeared by heat inactivation [26], [27], which suggests that protein components could be the key molecules of ASCs-E. Studies on pharmacokinetics and distribution of ASCs-E in vivo are also required for clinical application. To use ASCs-E on neurodegenerative diseases, measurement of blood-brain barrier permeability is needed. Future studies will help resolving these questions.

In summary, stem cell transplantation has been investigated as a therapeutic strategy to treat the HD. Considering clinical feasibility and safety, injection of ASCs-E could be effective and safe strategy for HD patients, comparable to the stem cell therapy. Findings from our study provide possible strategy to inhibit HD progression and reasonable resources for treatment.
